# No difference in subsequent trainee satisfaction associated with in-person exposure prior to remote interviews

**DOI:** 10.1080/10872981.2022.2122765

**Published:** 2022-09-08

**Authors:** Jocelyn Simmers, Nevada Cox, Beth Herman, Joslyn Kirby

**Affiliations:** a Department of Dermatology, Penn State Health, Hershey, PA, USA; b College of Medicine, Pennsylvania State University, Hershey, PA, USA; c Office of Graduate Medical Education, Penn State Health, Hershey, PA, USA

**Keywords:** in-person, virtual, interview, visit, exposure, trainee, resident, satisfaction

## Abstract

**Background:**

In 2020–2021, residency and fellowship applicants participated in virtual interviews. There was concern that trainees who had not been to the area before would potentially have different satisfaction with their new workplace and community.

**Objective:**

To compare satisfaction and likelihood to recommend work and community among new trainees with or without prior exposure to a single academic center or its environs.

**Methods:**

We conducted an IRB-approved cross-sectional survey of new trainees. An electronic survey included demographic items, self-report of prior exposure to the area, satisfaction with the program and area, and likelihood to recommend the program and area. Descriptive statistics were used for responses and Chi square tests for comparisons.

**Results:**

In September 2021 and May 2022 electronic surveys were sent to all 173 trainees who started residency or fellowship in July 2021, which had 87 responses (50.3% response rate) and 31 (18.0% response rate) responses, respectively. At both times, most respondents were interns. The majority of the September group (55.6%), while 38.7% of the May group had prior exposure to the area. Overall, the majority were satisfied with Penn State Health and would recommend their workplace. The majority also agreed they were satisfied with their new community and would recommend it to others. There were no significant differences in the proportions of satisfied trainees for any of the four outcomes at either timepoint.

**Conclusions:**

Satisfaction with training and the community were not significantly different for trainees with or without prior in-person exposure to the institution or surrounding area.

## Introduction

Institutions across the country implemented virtual interviews for residency and fellowship training programs in 2020–2021[1]. Literature shows mixed satisfaction with virtual interviews. Virtual interviews have been beneficial to applicants for financial purposes [2,3] and more convenient for applicants and interviewers alike [2,4]. In contrast, applicants reported virtual-only interviews negatively impacted their understanding of program culture [5,6]. Prior to the pandemic and widespread virtual interviews, more than 50% of matched applicants matched at their home program or a program where they completed an in-person rotation, so there is a concern that trainees’ satisfaction with their new training program would be negatively impacted due to a lack of prior in-person exposure. Physician satisfaction is important because dissatisfaction has been significantly associated with burnout and absenteeism as well as patient care [7–9]. The study objectives were to investigate differences in trainees’ satisfaction and likelihood to recommend the workplace and community, for trainees with or without prior exposure to the academic center or its environs.

## Methods

A cross-sectional, self-reported survey study was conducted in September to October 2021 and May 2022. The survey was developed, piloted, and data collected and managed using REDCap electronic data capture tools hosted at Penn State University [10]. REDCap (Research Electronic Data Capture) is a secure, web-based application designed to support data capture for research studies. An online link to the anonymous survey (Supplement) was sent to new residency and fellowship trainees after approximately 3 and 11 months from their start of training at an academic medical center. An invitation to complete the survey was sent from the staff from the Office of Graduate Medical Education, along with a reminder every five days and a maximum of four invitations. Upon completion of the survey, participants were offered a chance in a drawing to win a $50 gift card. Those who reported participation in-person interviews for their program were excluded.

The survey included demographic characteristics and confirmation the applicant had a virtual interview for the 2020–2021 cycle. Participants reported ‘exposure’ if they had any prior in-person exposure to the academic center or its environs (within 50 miles) with specific options including visitation for vacation or family, attended K-12, college, or medical school, or participated in a rotation during medical school or residency. Participants rated their satisfaction with their community and workplace as well as their agreement to recommend the institution and location to others. These outcomes of interest were assessed using four items, each with Likert scale response options.

All categorical survey data were presented as the number of subjects with percentages. Categorical and dichotomous variables were examined using the Chi square. Data analysis was conducted in June 2022 using SAS 9.4 software (SAS Institute Inc, Cary, SC). The Penn State University institutional review board determined this study as exempt.

## Results

Of the 173 new resident and fellowship trainees at our center, 50.3% (87/173) completed the survey, and 44.5% (77/173) responses met eligibility criteria in September and 18.0% (31/173) completed the survey and met eligibility criteria in May. Table 1 shows the demographic characteristics of respondents at both times. In September, the majority of respondents, 55.8% (43/77), reported prior exposure to the institution or area. Similarly, in May, the majority of respondents, 61.3% (19/31), reported prior exposure to the institution or area, specifically.

**Table 1.** Demographic characteristics and satisfaction of trainees over time.

	September			May		
Identified gender						
Male	43/76 (56.6%)			15/31 (48.4%)		
Female	32/76 (42.1%)			15/31 (48.4%)		
Non-binary	0 (0%)			0 (0%)		
Declined	1/76 (01.3%)			1/31 (3.2%)		
Race						
American Indian or Alaskan Native	0/77 (0%)			0/31 (0%)		
Native Hawaiian or other Pacific Islander	0/77 (0%)			0/31 (0%)		
Asian	27/77 (35.1%)			6/31 (19.4%)		
Black	1/77 (1.3%)			0/31 (0%)		
White	44/77 (57.2%)			20/31 (64.5%)		
Other	6/77 (7.8%)			5/31 (16.1%)		
Ethnicity						
Hispanic or Latino	4/76 (5.3%)			3/31 (9.7%)		
Not Hispanic or Latino	72/76 (94.7%)			28/31 (90.3%)		
Age						
<25	2/76 (2.6%)			0/31 (0%)		
25–29	48/76 (63.2%)			21/31 (67.7%)		
30–34	21/76 (27.6%)			7/31 (22.6%)		
35–40	4/76 (5.3%)			1/31 (3.2%)		
41–45	1/76 (1.3%)			0/31 (0%)		
46–50	0 (0%)			2/31 (6.5%)		
PGY						
1	61/77 (79.2%)			25/31 (80.7%)		
2	0 (0%)			1/31 (3.2%)		
3	0 (0%)			0/31 (0%)		
4	14/77 (18.2%)			4/31 (12.9%)		
5	1/77 (1.3%)			1/31 (3.2%)		
6	1/77 (1.3%)			0/31 (0%)		
7	0 (0%)			0/31 (0%)		
	Prior Hershey Exposure	No Prior Hershey Exposure		Prior Hershey Exposure	No Prior Hershey Exposure	
	43	34	p-value	19	12	p-value
Satisfied with Penn State Health as a place to work	40/43 (93.0%)	27/34 (79.4%)	0.10	12/19 (63.2%)	10/12 (83.3%)	0.42
Would recommend Penn State Health as a good place to work	36/43 (83.7%)	26/34 (76.5%)	0.43	14/19 (73.7%)	9/12 (75.0%)	1.0
Satisfied with the community	36/43 (83.7%)	26/34 (76.5%)	0.43	11/19 (57.9%)	10/12 (83.3%)	.24
Would recommend this area as a good place to live	39/43 (90.7%)	28/34 (82.4%)	0.32	13/19 (68.4%)	10/12 (83.3%)	.43

In September, 88.2% (67/76) of trainees reported they were satisfied with their residency program as a place to work, and 80.5% (62/77) of trainees agreed or strongly agreed they felt at home in the community. Additionally, 81.6% (62/76) of trainees would recommend the area as a good place to live and 88.2% (67/76) would recommend the institution as a good place to work (Table 1). In May, 71.0% (22/31) of trainees reported they were satisfied with their residency program as a place to work and 74.2% (23/31) would recommend the institution as a good place to work. Also, 67.7% (21/31) of trainees agreed or strongly agreed they felt at home in the community and 74.2% (23/31) of trainees would recommend the area as a good place to live (Table 1). For both time points, there was only one significant difference in satisfaction or recommendation for those with or without specific types of prior exposure (schooling, rotations, or social visits) (Table 2). Specifically, a higher proportion of respondents without prior school attendance in the area would recommend the institution as a good place to work (85.7% v 50.0%, p = .05). Similarly, there was a trend toward significance for a higher proportion of this group to also be satisfied with the institution as a place to work (81.0% v. 50.0%, p = 0.09).

**Table 2.** Trainee satisfaction and recommendation stratified by specific types of prior exposure to the area.

September	Attended school (K-12), college, and/or medical school			Participated in away rotation or elective in medical school or residency			Came to visit family or for vacation		
	Yes	No	p-value	Yes	No	p-value	Yes	No	p-value
Satisfied with Penn State Health as a place to work	11/12 (91.7%)	56/65 (86.2%)	.84	4/5 (80.0%)	63/72 (87.5%)	.51	29/32 (90.6%)	38/45 (84.4%)	.87
Would recommend Penn State Health as a good place to work	12/12 (100.0%)	50/65 (76.9%)	.99	3/5 (60.0%)	59/72 (81.9%)	.25	26/32 (81.3%)	36/45 (80.0%)	.89
Satisfied with the community	12/12 (100.0%)	50/65 (76.9%)	.99	5/5 (100.0%)	57/72 (79.1%)	.99	25/32 (78.1%)	37/45 (82.2%)	.65
Would recommend this area as a good place to live	10/12 (83.3%)	57/65 (87.7%)	.49	4/5 (80.0%)	63/72 (87.5%)	.51	29/32 (90.6%)	38/45 (84.4%)	.87
**May**									
Satisfied with Penn State Health as a place to work	5/10 (50.0%)	17/21 (81.0%)	.09	0	22/31 (71.0%)	na	9/12 (75.0%)	13/19 (68.4%)	.79
Would recommend Penn State Health as a good place to work	5/10 (50.0%)	18/21 (85.7%)	.05	0	23/31 (74.2%)	na	9/12 (75.0%)	14/19 (73.7%)	.67
Satisfied with the community	6/10 (60.0%)	15/21 (71.4%)	.4	0	21/31 (67.7%)	na	6/12 (50.0%)	15/19 (79.0%)	.10
Would recommend this area as a good place to live	6/10 (60.0%)	17/21 (81.0%)	.21	0	23/31 (74.2%)	na	10/12 (83.3%)	13/19 (68.4%)	.91

## Discussion

This study shows that new trainees, both those who had prior exposure to the area, and those who did not, had no statistical difference in their overall satisfaction with their residency program and location. Put another way, new trainees, who had not been to the institution or location prior to beginning their training were not less satisfied with the program than those who had been to the location. The one significant comparison in the study and one that trended toward significance showed that satisfaction and likelihood to recommend were higher in the group *without* prior schooling at the institution or in the area.

These findings present more support for virtual interviews by showing that regardless of in-person exposure to the residency program, new trainees are likely to be satisfied, and recommend the program all the same. Though studies have been conducted on satisfaction with online residency interviews [2,4,6,10], there is no previous literature on new residents who had virtual interviews, who have been to the location of their program before, versus those who have not, and if there is an association of overall satisfaction between either group.

The results of this study should be taken in the context of the limitations. This is a single-center study that assessed participants at two timepoints. Additionally, the study included a limited sample of trainees with the perspective of not having experience with the site prior to matching. There was also a limited response rate at the second timepoint. The experience of the residents at other locations may differ. There is also potential for measurement error, as we did not define locations within a 50-mile radius for participants taking the survey.

Future steps to take could include evaluating satisfaction between new resident cohorts who did in-person interviews versus those who did virtual interviews to further explore how in-person impacts overall satisfaction. Also, assessing how certain features of a virtual interview, such as pre-interview socials with current residents [2,3] or provision of pre-interview materials [11], may enhance overall satisfaction with both the interview and program.

## Conclusion

Our study shows that new trainees who had virtual interviews were satisfied with their program and would recommend the program, and the area to others, whether or not they had been to the area prior to residency for other reasons. While this is a negative study, it shows there was no difference among those with or without prior in-person experience and addresses one of the concerns that faculty may have about virtual interviews for residency and fellowship training programs. This may help programs as they consider utilizing virtual interviews.

## Supplementary Material

Supplemental Material

## Declarations

This is an observational study. The Pennsylvania State University Institutional Review Board has confirmed that no ethical approval was required.
