# Supplementary material for: No difference in subsequent trainee satisfaction associated with in-person exposure prior to remote interviews
Source: Med Educ Online. 2022 Sep 8;27(1):2122765. doi: 10.1080/10872981.2022.2122765 (PMC9467566; doi:10.1080/10872981.2022.2122765)
Supplement: Supplemental Material [file ZMEO_A_2122765_SM2159.docx]

Supplement. Invitation to participate and survey.

Hershey Location Satisfaction

Hello all,

When you matched at Penn State, you started a new position and joined a new community, with new places to live and eat as well as things to do. We want to learn more about your satisfaction with your work, workplace, and community.

If you participate you can be entered into a drawing for a $50 Amazon gift card! To award the card, we'll need your email address, but your responses will be anonymous. Every answer helps us!

Thanks,

The GME Team

To be eligible for the $50 Amazon gift card, please enter your email (this will be removed from your responses):

Please select your age range:

<25 years 25-29 years 30-34 years 35-40 years 41-45 years 45-50 years

Please select your current PGY year:

PGY-1 PGY-2 PGY-3 PGY-4 PGY-5 PGY-6 PGY-7

What gender do you identify as?

Male Female Non-binary Decline to answer

Please specify your race (Select all that apply):

American Indian or Alaska Native Asian Black or African American Native Hawaiian or Other Pacific Islander White Other/Unknown

Please specify your ethnicity:

Hispanic or Latino or Spanish Origin Not Hispanic or Latino or Spanish Origin

Did you have a virtual interview during July 2020 to January 2021 for your current training program?

Yes No

Did you have any of the following exposures to Hershey, PA before the start date of your current training program? (Select all that apply)

I attended school (K-12), college, and/or medical school within a 50-mile radius of Hershey PA

I did an away rotation or elective in medical school or residency within a 50- mile radius of Hershey PA

I previously came to Hershey, PA (or the surrounding area) to visit family or for vacation

Other

How satisfied are you with Penn State Health as a place to work?

Very satisfied Satisfied Neither satisfied/dissatisfied Dissatisfied Very dissatisfied

I feel at home in the community where I live now:

Agree Somewhat agree Neither agree or disagree Somewhat disagree Disagree

I would recommend this area as a good place to live:

Agree Somewhat agree Neither agree or disagree Somewhat disagree Disagree

I would recommend Penn State Health as a good place to work:

Agree Somewhat agree Neither agree or disagree Somewhat disagree Disagree
